# How Industry Uses the ICMJE Guidelines to Manipulate Authorship—And How They Should Be Revised

**DOI:** 10.1371/journal.pmed.1001072

**Published:** 2011-08-09

**Authors:** Alastair Matheson

**Affiliations:** Independent Consultant, London, United Kingdom and Toronto, Canada

## Abstract

Based upon his 15 years of experience as a medical writer, Alastair Matheson argues that rather than obstructing industry, the current ICMJE authorship guidelines have become its preferred tool for misattributing authorship.

**Summary Points**

- Academic authorship boosts the credibility of industry publications and masks their commercial function.
- Alongside traditional “guest authorship” and ghostwriting, industry may simply exaggerate the contribution of named academic authors and downplay that of commercial writers, who are excluded from authorship but listed as contributors in the small print.
- Rather than obstructing industry, the current International Committee of Medical Journal Editors (ICMJE) authorship guidelines provide a ready tool for misattributing authorship. Industry also relies on selective interpretations of key authorship concepts.
- The ICMJE guidelines should be fundamentally revised and the concept of origination given comparable importance to authorship and contributorship.
- Companies and writers who work on industry publications should be listed as byline authors.

## Introduction

Scientists and clinicians need to know the authorship, author interests, and origination of the articles they read to judge them appropriately. Since 1985, the International Committee of Medical Journal Editors (ICMJE) has provided evolving guidance on how authorship should be managed in the complex setting of modern biomedical science [1],[2], to the benefit of the published literature. Issues such as accountability, fraud, conflicts of interest, trial registration, and access to data have been considered by this voluntary, self-funded, closed-membership group of select general medical journal editors (http://www.icmje.org/) [3]–[5]. However, certain industry practices, including publications planning, ghostwriting, and guest authorship, have yet to be adequately addressed. On the basis of industry publications and documents, textual analysis, and direct working experience in the “medical communications” sector, I show here how pharma has succeeded not merely in outmaneuvering the ICMJE guidelines, but is able to use them as the basis for inappropriate attributions of authorship.

## Commercial Origination

Industry trials and publications have scientific but also commercial functions. In its dealings with academia, industry takes the misguided view that it should exaggerate the former and conceal the latter. Effective publications planning based on this premise enables industry to exert substantial control over the literature on its products and configure understandings of medicine such that their use seems reasonable [6]–[10]. Industry has realized that origination is a key determinant of how publications are perceived, and one that current guidelines do not adequately address. Accordingly, while collaboration between industry and academia can have benefits, academic authors groomed as “key opinion leaders” (KOLs) [6],[11] may be used not only to endorse publications, but also to convey the impression the publications were originated by academics. “Medical communications” agencies bear joint responsibility for these practices, and for the systematic masking of corporate origination within the medical literature. Industry claims its activities are ethical, but this is disingenuous and rests on two subtle strategies: first, the use of weak definitions or convenient understandings of concepts such as accountability, responsibility, authority, intellectual contribution, contributorship, guest authorship, and ghostwriting; and second, the exploitation of flaws in current guidelines, particularly those of the ICMJE.

## The Authorship-Contributorship Distinction Exploited

The important distinction between authorship and contributorship proves especially helpful to industry. Although contributorship was proposed as a replacement for authorship [12],[13], contributorship listings have acquired unintended parallels with advertising small print, and accordingly are used by industry to reduce its own visibility—despite, ironically, being used as the basis of claims to transparency. Through the exploitation of contributorship, crude ghostwriting and guest authorship are being replaced by more subtle exaggeration or understatement of authorial contributions. This practice is difficult to trace, since it involves subjective judgments, and the parties involved—companies, writers, and KOLs —all have incentives to allow their true levels of contribution to be aggrandized or downplayed. These practices gain succor from weak definitions of ghostwriting and ghost authorship, which the World Association of Medical Editors (WAME) and Council of Science Editors (CSE) deem not to have occurred if a writer is “mentioned in the manuscript” (WAME) or receives an “appropriate” place “in the author byline or Acknowledgments” (CSE) [14],[15]. Industry and medical writers' organizations are thus able publicly to condemn ghostwriting using comparable framings [16]–[18], while the misattribution of authorship remains widespread.

**Figure 1:**
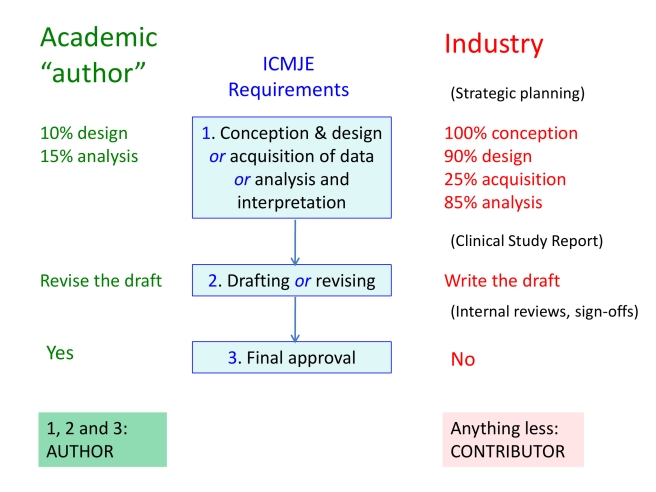
The ICMJE “triple lock” formula is a tool for industry. If final sign-off is ceded to the academic “author”, industry is disqualified from authorship even if it is responsible for most of the study and manuscript development. Manuscript writers are also disbarred even if they have sign-off, unless they were involved in other aspects of the study. These features give industry the opportunity to conceal its originating role behind the names of academic collaborators. Industry also carries out further originating activities not catered for in the ICMJE formula (shown in brackets). The percentage contributions shown here are for illustrative purposes only, and in reality vary widely.

From industry's perspective, the most useful feature of the current ICMJE guidelines is the formula used to distinguish between authors and contributors (Figure 1). To qualify as an author, an individual must (1) contribute substantially to either conception and design, or acquisition of data, or analysis and interpretation of data; *and* (2) draft the article or revise it critically for important intellectual content; *and* (3) be responsible for final approval of the manuscript [2]. This “triple-lock” formula has become a *de facto* license for misrepresentation. Provided academics make some contribution to design or data analysis, some revisions to a manuscript, and approve it, they are required to be named as authors. By contrast, industry may conduct most of the design, data collection and analysis, and all the writing, but if sign-off is ceded to the academic, it is disqualified from authorship. Unsurprisingly, the practice of ceding final sign-off to academic “authors” is widespread in commercially driven publications.

## Further Hazards of the “Triple-Lock”

The “triple-lock” formula also helps downplay the importance of planning and writing texts. Only in clause 2 is “drafting” acknowledged as a component of authorship, but since this clause can be satisfied by revision, it enables the planner and writer to be excluded. In reality, drafting constitutes a substantial intellectual contribution to the form and content of manuscripts. It is for this reason that industry seeks to control it, while evading the visibility of byline authorship. The “triple lock” provides ideal support for these linked objectives.

Industry finds further justification for self-concealment behind KOL “authorship” in the ICMJE requirement that authors should “take responsibility for appropriate portions of the content” of publications [2]. This statement and the “triple lock” are both attempts to implement the connecting concepts of accountability, responsibility, and guarantorship [5],[12],[19]. One difficulty with the current ICMJE wording on “responsibility” is that it emphasizes “content” alone, rather than all aspects of the publication. Furthermore, “content” can be narrowly interpreted by industry to omit the framings, nuanced constructions, and selectivity of data that are crucial to drug marketing [6],[9]. Moreover, “responsibility” and “accountability” are readily conflated by industry with notions of authority and expertise, such that it becomes legitimate for KOLs to assume responsibility for texts they neither originated nor wrote, while the contributions of the true originators are downplayed on the grounds that they lack the “authority” to stand for them. Writers and companies with the ability to generate academic texts should not be permitted to step back from responsibility on such disingenuous grounds; indeed, the true “authority” behind industry publications belongs to corporations, not their academic collaborators. Ultimately, industry's KOL-focused construction of “authority” is designed once again to downplay its own role—but also to appeal to the vanity of KOLs. Sadly, it is a construction that finds a ready reception within the culture of contemporary medicine.

Further aspects of current authorship practices provide support for industry. Notably, the ICMJE guidelines place great emphasis on the contributions of named individuals. This approach reflects traditional authorship customs, but assists unethical practices in two respects. Firstly, it helps entities, and in particular companies, remain concealed, particularly if their authorial role involves many individuals, each of whom is only a minor contributor to the finished publication. Secondly, it gives insufficient exposure to the process of origination by which publications are conceived and come into being—for commercially driven articles that exist to promote specific products, this information is vital to the reader.

Moreover, another deficiency in the current ICMJE guidelines concerns their policy on author access to data [5]. There is no requirement that any authors have permanent access to the study data or the right to re-analyze the data as they choose. Rather, authors need only have had access to the trial data at the time the study was conducted and the publication prepared. This is a weak position for the guidelines to adopt, whereas pharma, by contrast, asserts company ownership of the data in the trials “authored” by its KOLs [20].

In sum, the current ICMJE guidelines provide pharmaceutical and medical communications companies with the opportunity to sequester their contributions in the small print of publications, despite bearing responsibility for conception, design, and analysis of many studies, retaining control of databases, and frequently writing manuscripts, scheduling publications, and selecting journals. KOLs whose contributions may be modest and who lack full permanent access to the data may be the only individuals who qualify for authorship under ICMJE criteria. The current guidelines are therefore not an obstacle but a vehicle through which origination and authorship can be misrepresented to readers in the services of marketing, while enabling the companies involved to claim their conduct is compliant and ethical.

## Recommended Changes to Authorship Principles

For 26 years the ICMJE authorship guidelines have evolved by periodic adaptation, and in their current form have achieved broad support, notably from industry and commercial writers [20]–[23]. Clearly, however, a fundamental review is required. Indeed, such is the importance of the guidelines that the process by which they are devised and updated should itself be reviewed. In the meantime, guidelines developed by organizations involved with industry publications [21],[22] should not be recommended by journals to prospective authors. Furthermore, the commercially implanted phrase “industry-sponsored” trials (and publications), which is itself designed to downplay industry's role, should be replaced in the language of journals and medicine by the more truthful phrase “industry trials.”

**Figure 2:**
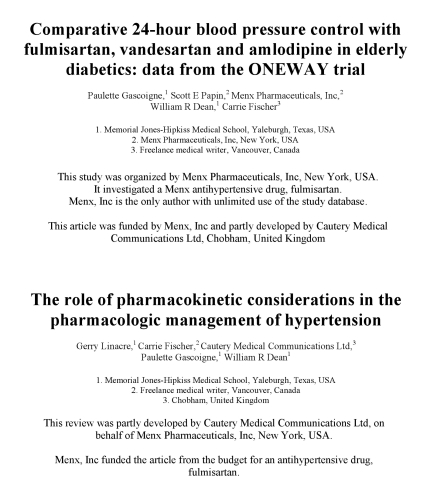
Examples of origination bylines and of entities as authors in journal articles.

With respect to the authorship issues discussed in this article, several philosophical clarifications are necessary. Firstly, while the categories of authorship, contributorship, and guarantorship remain important, comparable emphasis should be placed on the concept of origination, which differs from these categories in that it refers to a process rather than individual people. Some journals and indeed industry guidelines have made steps in this direction [24],[25], but these are insufficient. Crucial aspects of origination should be given immediate visibility: for instance, as depicted in Figure 2, companies and medical writers should be included among the named authors whenever appropriate, and both companies and specific drugs supported by the publication should be listed immediately below the author byline. This would enable readers immediately to recognize publications' commercial, as well as scientific, functions (Figure 2).

Secondly, it should be explicitly acknowledged that planning, drafting, and writing generally constitutes a significant intellectual contribution to a publication, and in most cases should require the individual and/or entity responsible to be listed as a byline author. This position has previously been advocated by Gøtzsche [23] and is implicit in statements by WAME and the policies of some journals [25]–[27]. Receiving input or “direction” from KOLs should not disbar writers from authorship. It should be emphasized that whenever writers are omitted from byline authorship by underplaying their true contribution, this constitutes ghostwriting, including when writers are listed as contributors.

Thirdly, greater provision should be made for authorship by entities, and in particular companies. Whenever an entity carries out activities that in the case of an individual would justify authorship, it should be listed as a byline author.

Fourthly, it should be clarified that responsibility and accountability pertain to all aspects of manuscripts, and all individuals and entities involved in developing them must be accountable. Responsibility for “content” should not be ceded to academic authors alone if others helped plan, write, or revise the manuscript. Academics may remain the only authors with the expertise to guarantee some aspects of content, but other key originators must also take visible responsibility (and credit) as byline authors.

In keeping with these principles, several specific measures should be considered:

1. With respect to the ICMJE guidelines, the “triple-lock” formula for distinguishing authors from contributors should be discarded. A model in which a variety of contributions require an individual or entity to be listed as an author should replace it.
2. For submissions in which companies or similar entities played any role in finance, planning, or development, a separate schedule (or supplement to the competing interests statement) should be completed to identify salient features of origination, including who planned and wrote the piece, which specific products the publication supported, and how it was financed. To ensure completeness, a company lawyer should be required to sign off. A further option would be to treat the agreement to publish as a legal contract, with accurate completion of the schedule a contractual condition.
3. Companies should be encouraged to provide full, permanent data access to academic authors. In all cases in which a company retained control or ownership of a trial database, the company itself should be required to be listed as one of the first three (and therefore cited) byline authors.

Finally, journal publishers and groups such as the ICMJE, CSE, and WAME should seek to establish links with bodies responsible for other aspects of medical-scientific discourse, such as continuing medical education (CME) organizations, professional societies, Web sites, and congresses, with a view to establishing an integrated, international standard of transparency in science [6]. Such a standard should require truthful, prominent display of salient origination and authorship. Academics would be able to place greater trust in articles, presentations, and CME courses bearing the standard's logo, and exercise appropriate caution with those which did not.

The ICMJE guidelines will always be a work in progress, but the adjustments proposed here have the potential to end the self-concealment and authorial misrepresentations that mar industry's contributions to the literature. Furthermore, they have the potential to help industry achieve the enhanced respect its beneficial contributions to medicine deserve. Industry publications will always have a commercial valence alongside their scientific and medical content: this should henceforth be truthfully displayed, and no longer downplayed or concealed.

## Abbreviations

ICMJE: International Committee of Medical Journal Editors
KOL: key opinion leader

## References

[1] International Committee of Medical Journal Editors (1985). Guidelines on authorship.. BMJ.

[2] International Committee of Medical Journal Editors (2011). Uniform requirements for manuscripts submitted to biomedical journals: writing and editing for biomedical publications.. http://www.icmje.org/.

[3] Davidoff F, DeAngelis CD, Drazen JM, Nicholls MG, Hoey J (2001). Sponsorship, authorship and accountability.. CMAJ.

[4] De Angelis CD, Drazen JM, Frizelle FA, Haug C, Hoey J (2005). Is this clinical trial fully registered? A statement from the International Committee of Medical Journal Editors.. Lancet.

[5] Drazen JM, de Leeuw PW, Laine C, Mulrow C, DeAngelis CD (2010). Toward more uniform conflict disclosures--the updated ICMJE conflict of interest reporting form.. N Engl J Med.

[6] Matheson AD (2008). Corporate science and the husbandry of scientific and medical knowledge by the pharmaceutical industry.. BioSocieties.

[7] Fugh-Berman AJ (2010). The haunting of medical journals: how ghostwriting sold “HRT”.. PLoS Med.

[8] The *PLoS Medicine* Editors (2009). Ghostwriting: the dirty little secret of medical publishing that just got bigger.. PLoS Med.

[9] Sismondo S, Doucet M (2010). Publication ethics and the ghost management of medical publication.. Bioethics.

[10] Egilman D, Druar N (2011). Corporate versus public interests: community responsibility to defend scientific integrity.. International Journal of Occupational and Environmental Health.

[11] Moynihan R (2008). Key opinion leaders: independent experts or drug representatives in disguise?. BMJ.

[12] Rennie D, Yank V, Emanuel L (1997). When authorship fails. A proposal to make contributors accountable.. JAMA.

[13] Smith R (1997). Authorship is dying: long live contributorship.. BMJ.

[14] World Association of Medical Editors (2005). Ghost writing initiated by commercial companies.. http://www.wame.org/resources/policies#ghost.

[15] Council of Science (2009). White paper on promoting integrity in scientific journal publications.. http://www.councilscienceeditors.org/i4a/pages/index.cfm?pageid=3355.

[16] International Society for Medical Publication Professionals Issues and Actions Committee (2010). Professional medical writing.. http://www.ismpp.org/initiatives/Files/ISMPP_Ghost_Writing_vs_Professional_Medical_Writing.pdf.

[17] European Medical Writers Association Ghostwriting positioning statement.. http://www.emwa.org/Home/Ghostwriting-Positioning-Statement.html.

[18] American Medical Writers Association (2009). http://www.amwa.org/default.asp?Mode=DirectoryDisplay&DirectoryUseAbsoluteOnSearch=True&id=466.

[19] Ilakovac V, Fister K, Marusic M, Marusic A (2007). Reliability of disclosure forms of authors' contributions.. CMAJ.

[20] Pharmaceu Reasearch and Manufacturers of America (2009). Principles on conduct of clinical trials. Communication of clinical trial results.. http://www.phrma.org/sites/default/files/105/042009_clinical_trial_principles_final.pdf.

[21] Graf C, Battisti WP, Bridges D, Bruce-Winkler V, Conaty JM (2009). Research methods & Reporting. Good publication practice for communicating company sponsored medical research: the GPP2 guidelines.. BMJ.

[22] Jacobs A, Wager E (2005). European Medical Writers Association (EMWA) guidelines on the role of medical writers in developing peer-reviewed publications.. Curr Med Res Opin.

[23] Gøtzsche PC, Kassirer JP, Woolley KL, Wager E, Jacobs A (2009). What should be done to tackle ghostwriting in the medical literature?. PLoS Med.

[24] British Medical Journal (2011). Article submission. Provenance of articles.. http://resources.bmj.com/bmj/authors/authors/article-submission/submitting-an-article-to-the-bmj#provenance.

[25] World Association of Medical Editors (2007). Authorship.. http://www.wame.org/resources/policies#authorship.

[26] Neurology (2010). Information for authors.. http://www.neurology.org/misc/auth2.xhtml.

[27] Proceedings of the National Academy of Sciences (2011). Information for authors. Journal policies. Authorship.. http://www.pnas.org/site/misc/iforc.shtml#ii.

